# Direct detection of Marek’s disease virus in poultry dust by loop-mediated isothermal amplification

**DOI:** 10.1007/s00705-014-2157-5

**Published:** 2014-07-02

**Authors:** Grzegorz Woźniakowski, Elżbieta Samorek-Salamonowicz

**Affiliations:** Department of Poultry Viral Diseases, National Veterinary Research Institute, Partyzantów 57 Avenue, 24-100 Puławy, Poland

**Keywords:** Marek’s disease virus, Poultry dust, Direct detection, Loop-mediated isothermal amplification

## Abstract

Marek’s disease virus (MDV) is a serious concern for poultry production and represents a unique herpesvirus model. MDV can be shed by doubly infected chickens despite vaccination. The fully infectious MDV particles are produced in the feather follicle epithelium (FFE), and MDV remains infectious for many months in fine skin particles and feather debris. Molecular biology methods including PCR and real-time PCR have been shown to be valuable for the detection of MDV DNA in farm dust. Recently, loop-mediated isothermal amplification (LAMP) was found to be useful in the detection of MDV in feathers and internal organs of infected chickens. LAMP is also less affected by the inhibitors present in DNA samples. Taking into account the advantages of LAMP, direct detection of MDV DNA in poultry dust has been conducted in this research. The detection of MDV DNA was possible in 11 out of the 12 examined dust samples without DNA extraction. The DNA was retrieved from dust samples by dilution and incubation at 95 °C for 5 min. The direct detection of MDV DNA in the dust was possible within 30 min using a water bath and UV light. The results were confirmed by electrophoresis and melting curve analysis of the LAMP products. Our results show that LAMP may be used to test for the presence of virulent MDV in poultry farm dust without DNA extraction.

Marek’s disease virus (MDV; species *Gallid herpesvirus 2,* genus *Mardivirus*, family *Herpesviridae* [5, 21]) is one of the most contagious agents in poultry production. Consequently, Marek’s disease (MD) has a huge economic impact [15, 17, 22, 25]. Recently, increasing incidence of MD caused by virulent MDV strains with increased pathogenicity has been observed [2, 8, 22, 25, 27]. Initially, MDV that is present in poultry farm dust infects the host via the respiratory tract [5, 13, 21]. The virus is then transferred by the bloodstream in alveolar macrophages to B and T lymphocytes. After the primary infection stage, between 7 and 14 days postinfection (dpi), MDV may become latent in infected lymphoid cells [5], which proliferate in different parts of host, especially in the liver, spleen, kidney, proventriculus and ovaries. This leads to tumour formation after reactivation of the virus to the transformation stage [17, 21]. Most MDV transmission occurs in the fully productive stage of infection and takes place in the feather follicle epithelium (FFE) [5, 7, 12, 19]. Subsequently, the virus is then transferred to the environment as fine particles of skin and feather debris [3, 4, 7, 11]. The DNA of MDV can be detected in poultry dust as early as 7 dpi [3, 4, 11]. Infectious MDV can persist in dust particles for many months and therefore be a potential source of infection for the next flock of chickens. Vaccination against MD with live attenuated vaccines safeguards against its clinical form as well as against tumours [21, 25]. However, this does not exclude the possibility of superinfection with very virulent MDV (vvMDV) and shedding of the virus into the environment [5]. Poultry farm dust may be also a good source of MDV DNA in order to monitor any possible future outbreak of infection in the flock following decontamination of an affected farm [7, 11, 13, 19, 22].

Conventional detection methods of viral DNA in dust such as polymerase chain reaction (PCR) and real-time PCR require the extraction of nucleic acids [3, 4, 6, 9–12, 19, 22–24]. PCR-based techniques are dependent on laboratory equipment, including thermocyclers or complex real-time PCR systems. Recently, loop-mediated isothermal amplification (LAMP) has been described as a powerful and rapid tool for MDV detection in infected chickens [1, 26]. LAMP partially fulfils expectations as an ideal diagnosis method because it can be used without access to advanced laboratory equipment [16, 18]. The bottleneck in the LAMP procedure is the extraction of nucleic acid.

The objective of this study was to apply the LAMP method for direct detection of MDV in poultry farm dust without DNA purification from crude samples. This is the first report of a LAMP application used for the monitoring of MDV in poultry farm dust.

The standard 31/07 vv+MDV strain (GenBank accession number HQ204806.1) with a titer of 10^4.1^ TCID_50_ (8812 PFU) was used as positive control for the MDV LAMP. The strain was propagated in SPF chicken embryo fibroblasts (CEFs). The stock of the 31/07 strain was stored in liquid nitrogen at (−196 °C). The DNA of the 31/07 strain was extracted from 200 µL of the virus stock using a QIAamp Mini Kit (QIAGEN, Hilden, Germany) as described previously [27].

Dust samples (about 20 g each) were collected from twelve different farms where MD had previously been diagnosed in chickens vaccinated with FC126 HVT and CVI988/Rispens. The dust from the farms was collected into plastic sealed bags, mainly from air fans, window sills and floors. The dust from each farm was then pooled, and the dust samples were then stored at −20 °C for further examination. About 20 mg of each pooled dust sample was resuspended in 480 µl of phosphate-buffered saline (PBS) by vigorous vortexing in a 1.5-ml Eppendorf^®^ tube. The samples were incubated for 5 min at 95 °C and then centrifuged at 6000×g for 3 min (Mikro 22R, Hettich Zetrifugen, Tuttlingen, Germany). The supernatants were then transferred to fresh microtubes and diluted 10-fold in PBS. The DNA yield and purity were measured by determining the A_260_/A_280_ ratio using a Nanophotometer^TM^, P-Class (Implen, Westlake Village, CA, USA). Three pairs of primers complementary to the *meq* gene sequence of the RB1B very virulent plus MDV strain (GenBank accession number AY571783) were designed using Primer Explorer version 4 (NetLaboratory, Tokyo, Japan) (Table 1) [26]. The reactions were set up on ice in 0.2-ml OptiAmp® optical tubes (Applied Biosystems, Foster City, CA, USA). The LAMP volume was 15 μL and contained 7.5 µL of Isothermal Mastermix (OptiGene, Horsham, West Sussex, United Kingdom), 50 pmol each of inner primers FIP and BIP, 10 pmol each of outer primers F3 and B3, 25 pmol each of loop primers LF and LB, 1 µl of 1:10,000-diluted ROX passive reference dye (EurX, Gdansk, Poland), 1 µl of PCR-grade water (EurX, Gdansk, Poland) and 2 µl of DNA template. After incubation of the reaction mixtures in a water bath (Memmert, Schwabach, Germany) at 62.3 °C for 30 min, 0.5 µl of a 1:100 stock dilution of 10,000-fold concentrated SYBR Green I dye in DMSO (Invitrogen) was added to each sample. The samples were observed under UV illumination to detect green fluorescence in positive samples. At the same time, the reaction was run in an ABI 7500 system (Applied Biosystems, Foster City, CA, USA) at 62.3 °C for 40 cycles (1 min each). Next, melting curve analysis was conducted at temperatures ranging from 95 °C to 60.0 °C. The positive control was DNA from the 31/07 strain, and the negative control DNA was extracted from uninfected CEFs. Pictures were taken using a G5 camera (Canon, Ōta, Tokyo, Japan). The cycle threshold values (C_T_) were recorded and melting curve analysis was done using the software from the ABI 7500 system (version 2.0.1). LAMP amplicons were separated in 2 % agarose gels stained with GelRed^TM^ dye (Biotum, Hayward, CA, USA).

**Table 1:** Sequences of LAMP primers used for detection of MDV-1 in dust samples

Primer	Sequence (5′-3′)
F3	TTCCCTCTTCTGCCCTCC
B3	TCCTGTTCGGGATCCTCG
FIP	GTAAACCGTCCCCGGCGATG*TTTT*GGGCATCTTCCCTGCATTG
BIP	CTTTGTCCTGTTGGCCAGGCTC*TTTT*GACGAGCATAAAGCCTCTCC
LF	TACACGGCTCGGTAACAGGA
LB	CCACATCCGGCTCCGGAGCC

*TTTT* is a thymidine linker in the FIP and BIP primers

The primers were designed based on the *meq* gene sequence of the RB1B very virulent plus MDV strain (GenBank accession number AY571783), using Primer Explorer version 4 (NetLaboratory, Tokyo, Japan)

In this study, we describe the first direct detection of MDV in crude dust samples using LAMP. The previously described application of LAMP for MDV DNA detection was conducted using samples extracted from feather tips using commercial kits or phenol-chloroform based procedures [26]. Over the past few years, LAMP has been found to be a powerful method for detecting a number of viral pathogens of poultry and free-ranging birds [1, 18, 26]. However, the application of an inexpensive method for in-farm detection of viral genetic material has been limited by the necessity of obtaining purified nucleic acids [16, 18]. Therefore, the development of a ‘portable’ technique that can be used even when access to laboratory equipment is limited has not been fully accomplished. The DNA polymerases used for LAMP include *Bsm*, *Bst,* *Gsp*SSD polymerase [1, 16, 26]. The last of these has been known to be less prone to inhibition than the other. Therefore, we decided to examine whether the simple process of dilution and heat treatment of dust samples from MDV-contaminated farms would be sufficient to perform LAMP using *Gsp*SSD polymerase. Indeed, by dilution and incubation at 95 °C of dust samples collected from 12 different chicken farms, it was possible to retrieve from 7.9 to 27.5 ng of DNA per µL. However, the crude samples appeared to contain protein contamination, since the A_260_/A_280_ ratio ranged from 0.38 to 1.0 (Table 2). In spite of this, the LAMP was found to be resistant to contamination, as we obtained a positive signal for MDV, visible as green fluorescence in 11 out of 12 dust samples (Fig. 1). The one negative dust sample that was obtained was also extracted using a commercial DNA extraction kit. However in spite of this, the sample remained MDV negative, possibly due to the presence of inhibitors. Indeed, attempts to amplify MDV DNA from dust samples using conventional PCR also failed (data not shown). The reliability of the results was confirmed by the C_T_ values in the ABI 7500 system (Table 2) as well as gel electrophoresis and melting curve analysis (Fig.1B and C). The common melting temperature for all of the MDV dust samples examined was about 87.8 °C. Interestingly, the C_T_ values that were obtained did not seem to be dependent on the purity of the DNA used, suggesting that the LAMP assay is resistant to inhibitors present in crude dust samples. Recently, Yoshikawa et al. [28] applied a dry-reagents LAMP assay for the direct detection of human herpesvirus 6B in human serum samples. They found the direct detection system to be a potentially perfect solution for major tropical diseases. Likewise, Segawa et al. [20] developed a direct LAMP-based detection technique for canine distemper virus (CDV), pseudorabies virus (PRV), feline calicivirus (FCV) and feline parvovirus (FPV) [20]. The authors used a RNA GEM Tissue Kit to extract mammalian DNA and RNA using heat treatment. A similar simplified DNA release method was used to detect *Aspergillus nomius* and *A. flavus* from samples of shelled Brazil nuts [14]. In the case of MDV, a good source of viral DNA is poultry dust. PCR and real-time PCR have been widely used for detection, monitoring and modelling of shedding patterns of MDV [2–4, 6, 9–12, 19, 23, 24]. The recently published study by Walkden-Brown et al. [24] showed that all three MDV serotypes could be detected in farm dust using quantitative real-time PCR. They found that 23.1 % of field dust samples contained DNA of MDV-1. The concentration of the extracted DNA was low and reached approximately 5 ng/µL, while the median value of the DNA purity reached 0.44, which supports the results obtained in our study (Table 2). Our results demonstrate the robustness of LAMP for MDV detection, even without DNA purification using crude dust samples. This procedure simplifies monitoring for the presence of MDV under farm conditions when access to laboratory equipment is limited.

**Table 2:** Detection of MDV DNA in dust samples by LAMP

Sample	DNA purity A_260_/A_280_ ratio/concentration (ng/µL)	Ct value LAMP MDV-1	Melting temperature
Negative control	1.83/125.0	40.0	-
Farm 1	0.47/24.3	30.4	87.4
Farm 2	0.38/15.9	18.3	86.9
Farm 3	0.41/19.0	21.4	86.8
Farm 4	0.42/20.5	15.4	87.4
Farm 5	0.45/27.5	40.0	-
Farm 6	0.41/19.9	16.7	86.9
Farm 7	0.45/20.1	20.5	87.4
Farm 8	0.41/19.4	20.7	87.3
Farm 9	0.39/7.9	14.0	86.5
Farm 10	1.0/12.7	20.9	86.8
Farm 11	0.46/18.4	17.0	87.1
Farm 12	0.40/10.5	15.9	86.3
Positive control DNA of 31/07 MDV strain	1.85/200.5	11.8	87.4

The measured DNA purity ratio (A_260_/A_280_), concentration (ng/µL), cycle threshold value and melting temperature of LAMP products are given. Negative control, DNA extracted from uninfected SPF chicken embryo fibroblasts (CEFs)

**Fig. 1:**
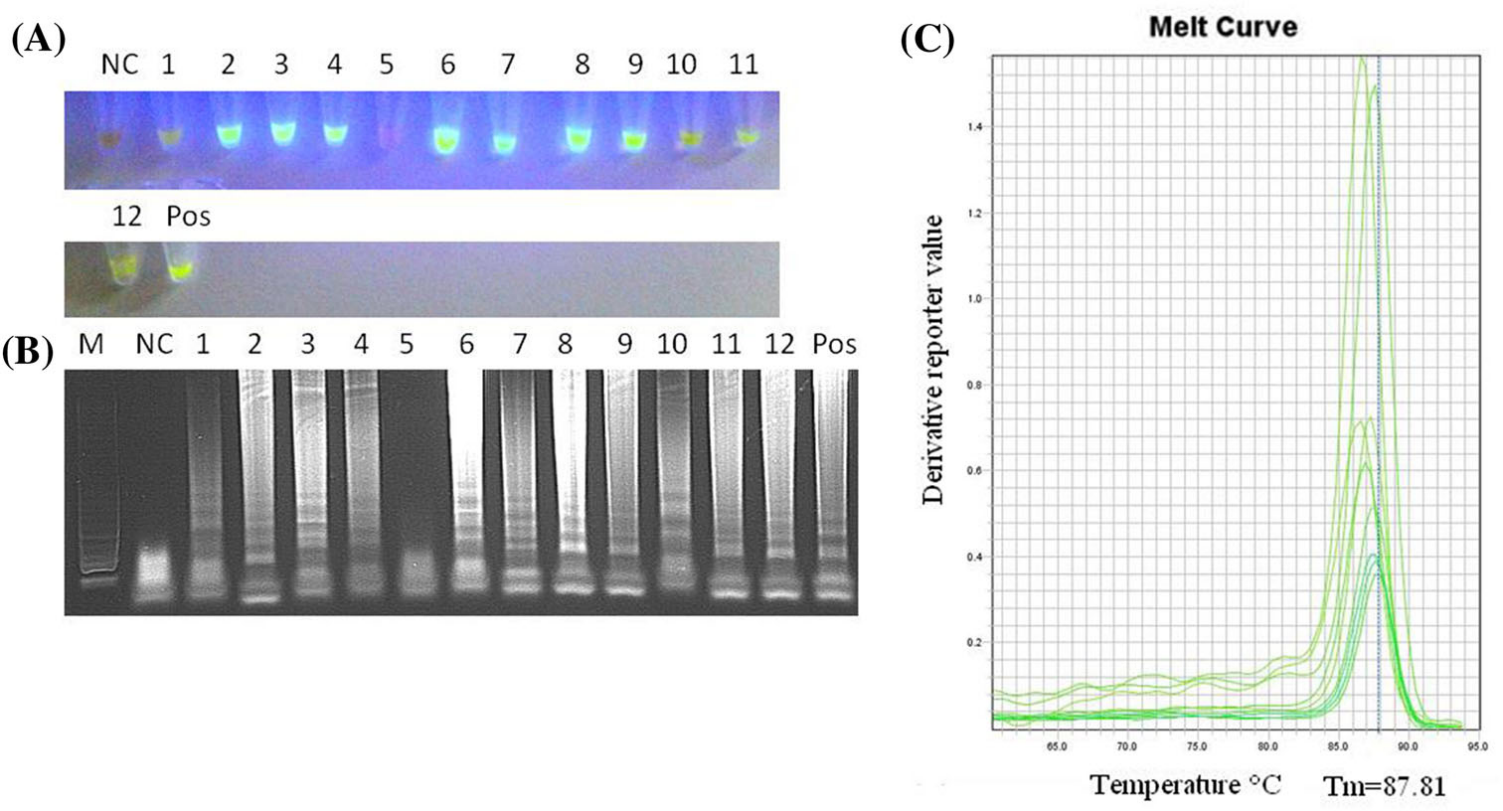
Direct detection of MDV DNA in poultry farm dust by LAMP. (A) Detection of MDV under UV light illumination after the addition of SYBR Green^®^ I dye, (B) electrophoresis of LAMP products in a 2 % agarose gel stained with GelRed^TM^ dye (Biotum, Hayward, CA, USA), (C) melting curve analysis of LAMP products conducted in a real-time PCR 7500 system (Applied Biosystems, Foster City, CA, USA). NC, negative control DNA extracted from uninfected SPF chicken embryo fibroblasts (CEF); Pos, positive control DNA of the standard 31/07 vv+MDV strain (accession number: HQ204806.1); 1-12, poultry dust samples collected from potentially MDV-contaminated farms; M, molecular size marker (GeneRuler™ 100bp DNA Ladder Plus, Thermo-scientific, Waltham, Massachusetts, USA). The common melting temperature point for all LAMP products was 87.8 °C. The derivative reporter value is plotted on the y-axis, whilst the temperature is plotted on the x-axis

In summary, this study shows that the use of simple diagnostic methods including simplified DNA extraction combined with LAMP may aid in the monitoring of chicken farm contamination with MDV. This potentially has an economic aspect, taking into account the high rate of transmission of MDV through poultry dust and the losses caused by MD.

In the future, it would be interesting to extend our study to LAMP detection of the remaining two MDV serotypes and attenuated CVI988/Rispens strains in crude dust samples.
